# Using SNP array data to test for host genetic and breed effects on Porcine Reproductive and Respiratory Syndrome Viremia

**DOI:** 10.1186/1753-6561-5-S4-S28

**Published:** 2011-06-03

**Authors:** Stefano Biffani, Sara Botti, Stephen C Bishop, Alessandra Stella, Elisabetta Giuffra

**Affiliations:** 1 Parco Tecnologico Padano-Loc. Cascina Codazza, Via A. Einstein, 26900 Lodi Italy; 2 The Roslin Institute and R(D)SVS, University of Edinburgh, Roslin, Midlothian EH25 9PS Scotland, UK; 3 Instituto di Biologia e Biotecnologia Agraria - CNR, Loc. Cascina Codazza, Via A. Einstein, 26900 Lodi, Italy; 4 Current address: INRA, UMR 1313 de Génétique Animale et Biologie Intégrative, Jouy-en-Josas, France – and - AgroP arisTech, UMR 1313 de Génétique Animale et Biologie Intégrative, Jouy-en-Josas, France

## Abstract

**Background:**

The effect of breed on Porcine Reproductive and Respiratory Syndrome Viremia (PRRSV) was tested using data collected in 17 Italian commercial pig farms and 1096 genotypes obtained by the PorcineSNP60 BeadChip. A binomial logistic model was used to investigate the relationship between breed-clusters and PRRSV susceptibility. Breed-clusters were defined using the matrix of genomic kinship between all pairs of piglets.

**Results:**

Only the contemporary group effect, defined as all piglets reared in the same herd, in the same year and whose samples were collected in the same season, was significant. Sex, age and breed-cluster showed no statistically significant effect on PRRS viremia, although the Landrace and Cross breed-clusters showed the lowest Odds-Ratio

**Conclusions:**

The model failed to detect a significant breed-cluster effect, highlighting the impact of environment and management on PRRS viremia incidence. Incomplete exposure over the observed period may have masked possible breed differences.

## Background

Over the last decade the genetics of host susceptibility/resistance to disease has been a major topic of research. For the swine industry PRRS represents one of the most economically important diseases worldwide, causing reproductive failure, abortions, stillbirths, interstitial pneumonia and decreased growth rate [1]. Previous studies investigated the possible role which breed effect may have in determining resistant/susceptibility of pigs to PRRSV. One author [2] in an experimental “*in vivo”* study found difference between *Duroc*, *Meishan* and *Hampshire* while two authors [3,4] with an “*in vivo”* infection experiment using *Large White*/*Landrace* and *Hamphshire*/*Duroc* synthetic lines found the former being more resistant to the effects of the virus. Ait-Ali *et al*[5] studied the innate immune response to PRRSV infection “*in vitro”*, *using* used flow cytometry to analyse cells in bronchial alveolar lavage fluid (BALF) from five commercial pig lines. Macrophages from the *Landrace* line showed significantly reduced virus replication and poor growth of PRRSV than *Large Whi*te, *Pietrain* and other two synthetic pig lines. Whilst *“in vivo”* and “*in vitro”* experiment can possibly help in dissecting PRRS pathogenesis among breeds, these studies were usually based on relatively small numbers of animals. Moreover, they do not consider environment and management which actually play an important role in the host resistant/susceptibility to PRRSV. Using data collected in commercial pig farms and genotypes obtained by the PorcineSNP60 BeadChip (Illumina, San Diego, CA), the objective of the present study was to cluster animals based on the average similarity among them and to test a possible breed-cluster effect on PRRS viremia.

## Methods

The data for the present study were extracted from an existing database belonging to the MISAGEN project [6], which included pedigree information, clinical symptoms, and health related phenotypes collected from a commercial pig breeding population in the north of Italy. The original dataset included records for the PRRS viremia measured by PCR in sera of 2908 weaning piglets from four breeds, namely *LargeWhite*, *Landrace*, *Duroc* and *Pietrain*. PRRS viremia was defined as a binary trait based on the results of the traditional PCR: negative samples were coded as 0, positive samples as 1. DNA samples from 1,096 piglets were genotyped for 64,232 SNPs using the PorcineSNP60 BeadChip [7]. Negative and positive piglets numbered 766 and 440 animals, respectively. The number of negative/positive piglets by breed is shown in table 1. Prior to statistical analyses genotype quality control was performed using the *quality control* function implemented in the GenABEL package for R statistical software [8]. The following filters were applied for exclusion of individual single nucleotide polymorphisms (SNPs):

**Table 1:** Number of negative (0) and positive (1) piglets by breed (original dataset)

Breed	0	1	% Infected
Duroc	189	137	42%
Landrace	134	67	33%
Large White	309	207	40%
Pietrain	24	29	55%

Total	656	441	40%

- call-rate (%) < 99 (if the SNP was available in less than 99% of all genotyped individuals)

- minor allele frequency (MAF) in all individuals < 0.05

Furthermore individuals <99% call rate (maximum percent of missing genotypes in an individual) were eliminated. A total of 14,967 SNPs (24.8%), from the available 60,123 SNPs, were excluded from the case-control analysis if one of the filters indicated a violation of the quality. A total of 77 (0.063%) individuals were excluded: 33 individuals who had low call rates, 3 individuals with too high Identity By State (IBS) and 41 individuals with sex discrepancies. A contemporary group (Herd-Year-Season, HYS) was defined as all piglets reared in the same herd, in the same year and whose samples were collected in the same season. Sampling season was categorized as season 1 (January to April), season 2 (May to August) and season 3 (September to December). Totally, 46 HYS groups were defined whose average size was 9.1. Three breeds were present in twenty HYS (43 %), two breeds in sixteen HYS (35%), four breeds in three HYS (15 %) and only one breed in seven HYS (15 %). A General Linear Mixed Model with a logit link function and binomial distributions was used to investigate the relationship between breed and PRRSV susceptibility using the Glimmix procedure in SAS version 9.1 (SAS Institute Inc., Cary, NC). Breed-clusters were defined using the matrix of genomic kinship between all pairs of piglets computed as:

, where L is the number of loci, p_l_ is the allelic frequency at l-th locus and g_l;j_ is the genotype of j-th individual at the l-th locus, coded as 0, 1=2, and 1, corresponding to the homozygous, heterozygous, and other type of homozygous genotype. Such a matrix was transformed to a distance matrix and the first two principal components were computed by Classical Multidimensional Scaling. The fitted model was: *Y_ijkl_* = *HYS_i_* + *G_j_* + *B_k_* + *F_l_* + *b.age_l_*+ *e_ijkl_* (1), where Y is the binary trait, HYS is the fixed effect of the ith contemporary group, G is the fixed effect of jth gender class, B is the fixed effect of the kth breed-cluster class, F is the random effect of lth piglet, age is the covariate for age at sampling, with b being the regression coefficient, and e is the random error.

## Results

**Figure 1:**
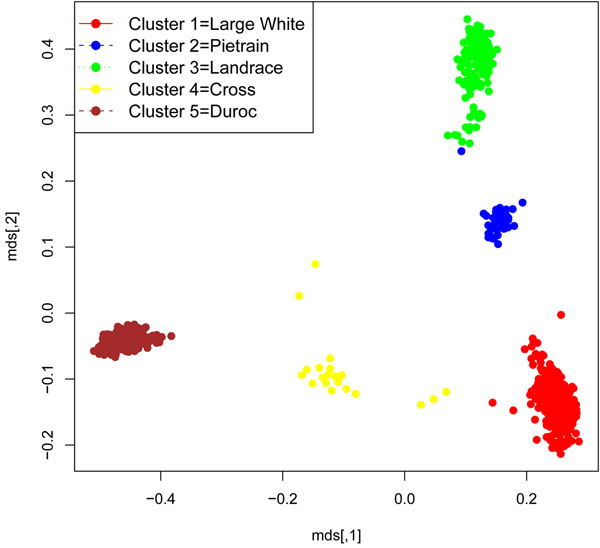
Multidimensional Scaling Plot – Breed Clustering

**Figure 2:**
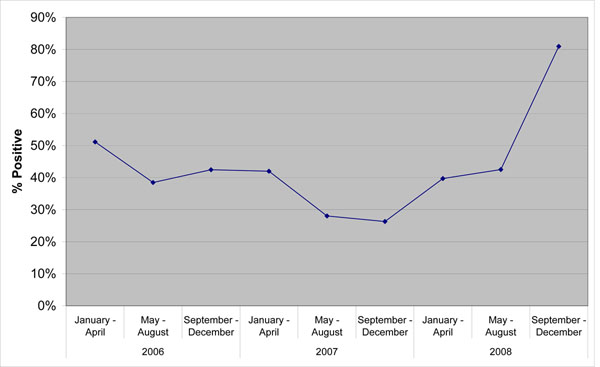
Observed PRRS viremia incidence over the test period

Breed-clusters as a result of "a posteriori" definition based on Kinship analysis are plotted in Figure 1. Each point represents a piglet. As expected, using SNP information, four main clusters can be identified. Those clusters matched with the expected breed, defined by the time of field data recording. However, an additional cluster (cluster 4) can be identified between cluster 1 (*Large White*) and cluster 5 (*Duroc* ). Those animals are possibly the result of cross-breeding. Indeed, they belong to farms where both breeds (i.e, *Large White* and *Duroc*) were present and where artificial insemination was used. Our hypothesis is that some inconsistencies occurred during the insemination events, *i.e.* that a limited number of boars was associated to the wrong insemination. According to the breed-cluster classification (Table 2), the observed proportion of affected piglets ranges from 34% (*Landrace* – Cluster 3) to 52% (*Pietrain* – Cluster 2). Results from Type III test of fixed effects are shown in table 3. Only the Herd-Year-Season effect (contemporary group) is significant. Sex, age and breed-cluster show no statistically significant effect on PRRS viremia, although the *Landrace* and Cross clusters show the lowest Odds-Ratio (not shown). Results on breed-cluster effect are contradictory. Some studies reported the existence of differences in susceptibility among breeds [9,10]. Nevertheless, most of those studies were based on in vitro or in vivo experiments, used commercial data from few large farms or analyzed different phenotypes. The present study spanned a 3-year period and used data collected from 17 farms. As Cooper et al. [11] observed, data under controlled experimental conditions do not necessarily support field reports. Physical and environmental factors may affect the immune system and hence determine or change the host response. This is exactly what our results suggest, because the contemporary group effect tries to capture the combined effect of environment and management removing variation due to their conditions over time. Previous findings from MISAGEN data [12] do show the existence of genetic variation but no breed effect. In this case breed was not defined by Kinship analysis. Bishop and Woolliams [13] showed how incomplete exposure to infection can reduce the power of datasets. This is the case of the present study, where records were collected in commercial farms spanning a 3-year period. As can be observed in Figure 2 incidence varies from a minimum of 27 % (September – December 2007) to a maximum of 80 % (September – December 2008).

**Table 2:** Number of negative (0) and positive (1) piglets by breed-cluster (after SNP editing)

Breed-Cluster	0	1	% Infected
1 (Large White)	271	182	40%
2 (Pietrain)	26	28	52%
3 (Landrace)	121	63	34%
4 (Cross)	10	10	50%
5 (Duroc)	180	128	42%

Total	608	412	40%

**Table 3:** Type III text of fixed effects of breed, age, HYS and sex

Effect	Num DF	Den DF	F Value	Pr > F
Breed-Cluster	4	317.6	0.91	0.4597
age	1	651.7	0.24	0.6216
hyseason	45	172.5	1.9	0.0018
sex	1	967	0	0.9815

## Conclusions

The idea behind this study was to use SNP to correctly cluster animals based on the average similarity among them and to test a possible breed-cluster effect using a General Linear Mixed model. The model fails to detect a significant breed-cluster effect but highlighted the impact of environment and management on PRRS viremia incidence. Although we cannot formally exclude that incomplete exposure over the observed period may have masked possible breed differences, the genome wide analysis currently in progress could detect a significant genetic variability in host response to PRRSV in the same dataset (Botti, Biffani et al. in preparation).

## Competing interests

The authors declare that they have no competing interests.

## Authors' contributions

STB and SAB wrote the manuscript. STB performed the statistical analysis and interpretation of data. SAB designed and coordinated the acquisition of genotypic and phenotypic data. SCB contributed to design and interpretation of data and critically revised the manuscript. ALS contributed to design and supervised the statistical analysis. ELG conceived of the study and coordinated it.

All authors read, revised and approved the final manuscript.

## References

[B1] Neumann EJ Kliebenstein JB Johnson CD Mabry JW Bush EJ Seitzinger AH Green AL Zimmerman JJ Assessment of the economic impact of porcine reproductive and respiratory syndrome on swine production in the US J 2005 227 385 392 10.2460/javma.2005.227.385 16121604

[B2] Halbur PG Rothschild MF Thacker BJ Meng XJ Paul PS Bruna JD Differences in susceptibility of Duroc, Hamsshire, and Meishan pigs to infection with a high virulence strain (VR2385) of porcine reproductive resiratory syndrome virus (PRRSV) J 1998 115 181 189 10.1111/j.1439-0388.1998.tb00341.x

[B3] Petry DB Holl JW Weber JS Doster AR Osorio FA Johnson RK Biological responses to porcine respiratory and reproductive syndrome virus in pigs of two genetic populations J Anim Sci 2005 83 1494 502 15956456 10.2527/2005.8371494x

[B4] Petry DB Lunney J Boyd P Kuhar D Blankenship E Johnson RK Differential immunity in pigs with high and low responses to porcine reproductive and respiratory syndrome virus infection J Anim Sci 2007 85 2075 92 10.2527/jas.2006-721 17468430

[B5] Ait-Ali T Wilson AD Westcott DG Clapperton M Waterfall M Mellencamp MA Drew TW Bishop SC Archibald AL Innate immune responses to replication of porcine reproductive and respiratory syndrome virus in isolated Swine alveolar macrophages Viral Immunol 2007 20 105 18 10.1089/vim.2006.0078 17425425

[B6] Botti S Caprera A Gaita L Mondin P Ossani N Palermo S Luini M Vezzoli F Cordioli P Nigrelli P Fallacara C Barbieri I Pacciarini M Bandi C Stella A Giuffra E The misagen project: towards the genetic improvement of disease resistance of pig commercial populations Proceedings 8th WCGALP 2006 26 414 417

[B7] Ramos AM Crooijmans RPMA Affara NA Amaral AJ Archibald AL Design of a High Density SNP Genotyping Assay in the Pig Using SNPs Identified and Characterized by Next Generation Sequencing Technology PLoS ONE 2009 4 8 e6524 doi:10.1371/journal.pone.0006524 10.1371/journal.pone.0006524 19654876 PMC2716536

[B8] Aulchenko YS Ripke S Isaacs A van Duijn CM GenABEL: an R library for genome-wide association analysis Bioinformatics 2007 23 1294 1296 10.1093/bioinformatics/btm108 17384015

[B9] Lewis CR Torremorell M Galina-Pantoja L Bishop SC Genetic parameters for performance traits in commercial sows estimated before and after an outbreak of porcine reproductive and respiratory syndrome J Anim Sci 2009 87 876 84 10.2527/jas.2008-0892 18952741

[B10] Reiner G Willems H Pesch S Ohlinger VF Variation in resistance to the porcine reproductive and respiratory syndrome virus (PRRSV) in Pietrain and Miniature pigs J Anim Breed Genet 2010 127 100 6 10.1111/j.1439-0388.2009.00818.x 20433517

[B11] Cooper VL Doster AR Hesse RA Harris NB Porcine reproductive and respiratory syndrome: NEB-1 PRRSV infection did not potentiate bacterial pathogens J Vet Diagn Invest 1995 7 313 20 7578444 10.1177/104063879500700303

[B12] Biffani S Botti S Caprera A Giuffra E Stella A Genetic Susceptibility to Porcine Reproductive and Respiratory Syndrome (PRRS) virus in commercial pigs in Italy Proceedings 9th WCGALP in press

[B13] Bishop SC Woolliams JA On the genetic interpretation of disease data PLoS One 2010 5 1 e8940 10.1371/journal.pone.0008940 20126627 PMC2812510

